# A novel chimeric *CYP11B2/CYP11B1* combined with a new p.L340P CYP11B1 mutation in a patient with 11OHD: case report

**DOI:** 10.1186/s12902-018-0249-z

**Published:** 2018-04-27

**Authors:** Lian Duan, Rufei Shen, Lingyu Song, Yong Liao, Hongting Zheng

**Affiliations:** 1 Department of Endocrinology, Xinqiao Hospital, Third Military Medical University, Chongqing, 400037 China; 2 Department of Endocrinology, Armed Police Hospital of Chongqing, Chongqing, 400061 China

**Keywords:** Chimeric *CYP11B2/CYP11B1*, Missense mutation, 11β-hydroxylase deficiency

## Abstract

**Background:**

11β-Hydroxylase deficiency (11OHD) is a common form of congenital adrenal hyperplasia that has been shown to result from inactivating *CYP11B1* mutations, and pathogenic *CYP11B2/CYP11B1* chimeras contribute to a minority of cases. Heterozygote cases (chimeras combined with missense mutation) are very rare, and genetic analysis of these cases is difficult.

**Case presentation:**

We describe an 11OHD patient presenting with precocious pseudopuberty and hypokalemia hypertension who harbored a chimeric *CYP11B2/CYP11B1* with a novel breakage point located at g.9559–9742 of *CYP11B2*. Interestingly, the other allele exhibited a new mutation, p.L340P, in *CYP11B1*. Bioinformatics and molecular dynamics simulation indicated that p.L340P decreased the stability and changed the surface configuration of 11β-hydroxylase, indicating a disease-causing mutation. Further pedigree study, PCR and next-generation sequencing indicated that the proband carried both the chimera and p.L340P, and coexistence of the two increased the severity of 11OHD in this family. After treatment with combined medications, blood pressure and clinical parameters improved.

**Conclusions:**

Our results suggest that chimera screening and *CYP11B1* mutation screening should be simultaneously conducted, and pedigree study is necessary.

**Electronic supplementary material:**

The online version of this article (10.1186/s12902-018-0249-z) contains supplementary material, which is available to authorized users.

## Background

Congenital adrenal hyperplasia (CAH) is one of the most common inheritable metabolic disorders and is characterized by virilization, precocious pseudopuberty and accelerated skeletal maturation, progressing in some cases to severe dehydration, shock, and even death [1, 2]. An autosomal recessive disorder, CAH is caused by mutations in genes encoding important enzymes or cofactors in the steroidogenesis pathway [3]. One common variant of CAH is 11β-hydroxylase deficiency (11OHD), driven by *CYP11B1* inactivating mutations clustered in exons 2, 6, 7 and 8 [4], and approximately 148 mutations have been reported in the Human Gene Mutation Database website. However, *CYP11B1* mutations may also occur as a result of aberrant incorporation/chimerism of the gene with the highly homologous aldosterone synthase (*CYP11B2*) gene sequence. These *CYP11B2/CYP11B1* chimeric genes are relatively rare, with breakpoints dispersively distributed, and have been speculated to be pathogenic because of a loss of function in the zona fasciculate/reticularis, despite maintained function in the zona glomerulosa [5–9]. In a few cases, missense mutation and chimera have been found in the same individual [6]. However, it is difficult to evaluate the precise contributing mechanism.

In the present paper, we report an 11OHD case who harbored a chimeric *CYP11B2/CYP11B1* on one allele with a breakpoint range (g.9559–9742) in the *CYP11B2* gene in the junctional zone of exon 6 and intron 6. Interestingly, the other allele held a novel mutation, p.L340P, and bioinformatics and molecular dynamics simulation indicated that this was a disease-causing mutation. We then performed a pedigree analysis, which revealed that both the missense mutation and chimera were synergistically pathogenic in the proband.

## Case presentation

**Fig. 1:**
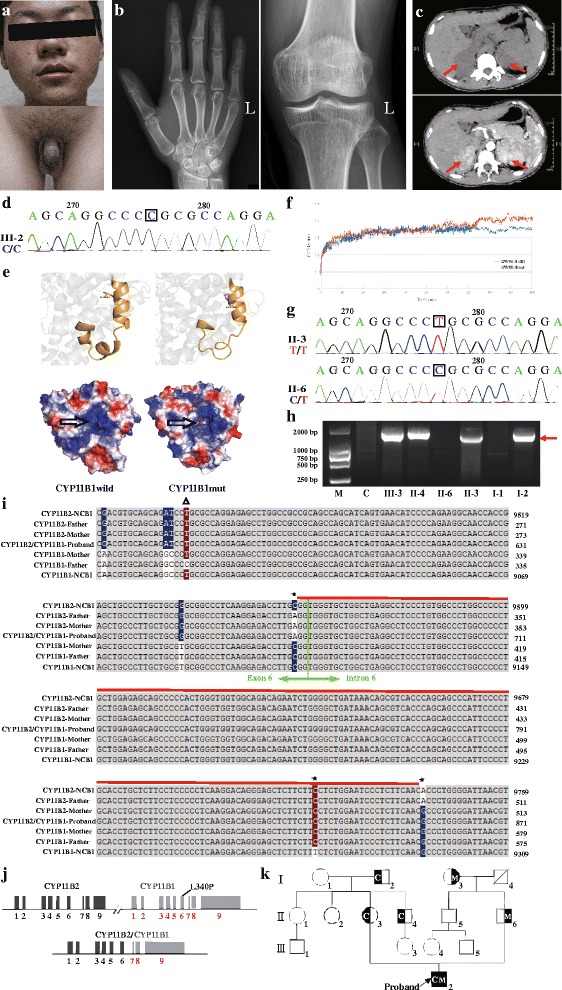
Clinical data and gene mutations of the proband and his family. **a** Physical examination results for the proband. **b** Radiograph of left wrist and knee. **c** Abdominal CT scan of the proband, with and without contrast enhancement. Arrow shows the bilateral adrenal lesions. **d** The sequencing chromatogram near the mutation in proband. Box indicates the mutation location. **e** Three-dimensional structure of wild-type *CYP11B1* (*CYP11B1wild*) and the *CYP11B1* mutant (*CYP11B1mu*t) after 100 ns simulation based on bioinformatics and the molecular dynamics simulation. **f** Chart of root mean square deviation (RMSD) calculations for both *CYP11B1wild* and *CYP11B1mut* in the molecular dynamics simulation. **g** The sequencing chromatogram on both sides of the mutation in the proband’s parents. The box indicates the mutation location. **h** Electrophoretogram (on a 1% agarose gel) of PCR products obtained from control, proband and proband relatives using mixed primers. The arrow shows the 1649 base pair DNA fragment of the *CYP11B2/CYP11B1* chimera. **i** The nucleotide sequence neighboring the crossover site of the chimeric *CYP11B2/CYP11B1* gene based on multiple sequence alignment. The asterisk, triangle and red line represent a single nucleotide polymorphism, p.L340P and the possible breakpoint regions, respectively. **j** A schematic representation of genotype for the proband, the *CYP11B2* gene (exons displayed as black boxes), the *CYP11B1* gene (exons displayed as gray boxes) and the hybrid gene with the breakpoint localized in the junctional zone of exon 6 and intron 6. **k** The pedigree of all family members investigated for the missense mutation (M) and the chimeric gene (C)

A 14-year-old boy (46, XY) was admitted in September 2015 because of precocious puberty and recurrent episodes of periodic paralysis without special intervention. His sexual development, such as pubic hair, laryngeal prominence and spermatorrhea, occurred earlier than that in peers. He was born after an uneventful full-term pregnancy to a non-consanguineous healthy couple of Chinese origin with no family history of congenital adrenal hyperplasia. He was taller than boys of the same age until he was 12 years old. In addition, axillary hair and laryngeal prominence had appeared at 7 years old, and spermatorrhea and pubic hair growth had occurred at age 9. Physical examination showed hypertension (143/106 mmHg), slightly black skin, and external genitalia maturation (pubic hair and axillary hair at stage 5 assessed by Tanner classification and an estimated testicular volume of 25 ml on both sides, as measured by an orchidometer), which confirmed precocious puberty (Fig. 1a). Laboratory data in the absence of medication showed decreased plasma potassium and cortisol but elevated levels of plasma 17 hydroxyprogesterone (17OHP), androstenedione, adrenocorticotrophic hormone (ACTH), and uric 17 ketosteroid (17 KS) (Additional file 1: Table S1). Additionally, serum aldosterone (ALD) was normal with low rennin activity, and renal, liver, metadrenaline, normetadrenaline and thyroid function, were all normal (Additional file 1: Table S1). Radiation imaging showed accelerated bone aging (over 18 years according to the Greulich and Pyle Atlas) (Fig. 1b) and bilateral adrenal hyperplasia (Fig. 1c). The hypokalemic hypertension, elevated 17-OHP and androstenedione were all suggestive of an 11OHD diagnosis. Interestingly, low urine osmotic pressure and specific gravity could not be significantly elevated by water deprivation and a desmopressin test. This phenomenon is consistent with nephrogenic diabetes insipidus that may be due to long-term hypokalemia (Additional file 1: Table S1).

Given this clinical presentation, we then sought to confirm whether it was indeed a case of 11OHD. To find the possible genetic pathogenic mechanism, we conducted sequencing of the nine exons and flanking sequencing of the *CYP11B1* gene and identified the novel mutation p.L340P (*CYP11B1mu*t) (Fig. 1d). Bioinformatics and molecular dynamics simulation indicated that *CYP11B1mut* altered the free energy, and change the stability and conformation of the protein by driving the uncoiling of a partial helical structure into a loop structure, leading to positive group migration and pocket structure enlargement (Additional file 2: Table S2, Figure 1e, f), suggesting that the mutation could be pathogenic. However, his mother did not carry this pathogenic mutation (Fig. 1g), indicating the proband only carried the point mutation on one allele, with the pathogenicity of the other allele unclear. Since 11OHD is understood to be an autosomal recessive condition, we then investigated the genotype of the patient in greater detail. QPCR of the sequence around the mutation of the proband showed that the copy number was half the normal number, which indicated a fragment deletion, and this was also found in his mother (Additional file 3: Figure S1). Targeted next-generation sequencing of the patient genome then revealed a large fragment deletion that included exons 1 to 6 of *CYP11B1* (Additional file 3: Figure S1). To validate the deletion, we conducted PCR in proband with mixed oligonucleotide primers that have been previously reported [8] (a forward primer complementary to the *CYP11B2* sequence and a reverse primer complementary to the *CYP11B1* sequence) and confirmed a chimeric *CYP11B2/CYP11B1* gene. Interestingly, extended familial analysis revealed that the chimera was also present in his mother, uncle and grandfather (Fig. 1h), and multiple sequence alignment revealed that the breakpoint was located at g.9559–9742 of *CYP11B2,* considering the cytosine nucleotide base (C) of rs6391 in this family with 3 single nucleotide polymorphisms (SNPs) (Fig. 1i). Our results suggested that the proband harbored a compound heterozygosity for a chimeric *CYP11B2/CYP11B1* gene combined with a novel missense mutation p.L340P (Fig. 1j and k**,** Additional file 4: Figure S2) as two contributors to pathogenicity.

Since the blood pressure of the patient was poorly controlled by hydrocortisone, the proband was treated with a combination of dexamethasone (0.75 mg/everyday) and levamlodipine (2.5 mg/everyday) for 20 months. After treatment, clinical parameters, such as blood pressure, 17OHP and ACTH decreased, and the volume of the adrenal glands was reduced (Additional file 1: Table S1, Additional file 5: Figure S3). Main methods used in the study are summarizing in Additional file 6. This study was approved by the ethics committees of Xinqiao Hospital, Third Military Medical University, and informed consent was obtained from the patient’s mother (Additional file 7).

## Discussion and conclusion

11OHD presents with decreased cortisol and corticosterone synthesis due to impaired conversion of 11-deoxycortisol and 11-deoxycorticosterne (DOC) to cortisol and corticosterone, respectively, and excess androgens due to DOC accumulation [10]. Although DOC was not detected, all of the classical 11OHD symptoms, precocious pseudopuberty, accelerated skeletal maturation and hypokalemia hypertension [11, 12], were present in our patient. After treatment with the combined medication regimen described above, the clinical symptoms of the patient improved and the adrenal gland volume was reduced.

From our investigation of the genetic contribution to pathogenesis, this patient was found to harbor a chimeric *CYP11B2/CYP11B1* gene with a novel breakpoint (g.9559–9742) on one allele, which differs from previous reports. Interestingly, the other allele contained a new disease-causing mutation, p.L340P, which altered the free energy and stability of 11β-hydroxylase. The chimeric mutation leads to deletion of CYP11B1 exons 1 to 6 on one allele, and unfortunately, a pathogenic missense mutation appeared on exon 6 of CYP11B1 in the other allele, which resulted in a seemingly homozygous missense mutation that was predicted by bioinformatics and molecular dynamics simulation to be a disease-causing mutation. At present, only one case of a pathogenic p.G314R mutation combined with a nonfunctional chimeric *CYP11B2/CYP11B1* has been reported [6]. Our pedigree analysis revealed that the proband carried both the chimera and p.L340P, and their coexistence increased the morbidity of 11OHD in this family. Our results suggest that chimera screening and *CYP11B1* mutation screening should be simultaneously conducted and that pedigree study is necessary.

## Additional files

Additional file 1: **Table S1.** The laboratory and endocrinological evaluation of the proband pre- and post-treatment. (PDF 200 kb)

Additional file 2: **Table S2.** Bioinformatics analysis of the free energy alteration in CYP11B1mut (PDF 82 kb)

Additional file 3: **Figure S1**. Graphs of sequence copy number around the mutation and multiple genes resulting in CAH of the proband and his mother by qPCR and targeted next-generation sequencing. (TIF 11910 kb)

Additional file 4: **Figure S2.** The sequencing chromatogram near the mutation in the proband’s relatives. The box indicates the mutation location. (TIF 28380 kb)

Additional file 5: **Figure S3**. Radiation imaging of adrenal gland after treatment. A and B present abdominal CT images after treatment for 6 months showing that the sizes of the left and right adrenal gland are 75.2 mm × 22.1 mm and 67.3 mm × 38.7 mm, respectively. Similarly, C and D present abdominal CT images after treatment for 9 months showing that the sizes of the left and right adrenal gland are 65.2 mm × 24.4 mm and 63.7 mm × 35.7 mm, respectively. The arrow shows the bilateral adrenal lesions. (TIF 63145 kb)

Additional file 6: Main methods used in the study. (DOC 112 kb)

Additional file 7: Consent form from the mother of the proband. (PDF 95.7 kb)

## Abbreviations

11OHD: 11β-hydroxylase deficiency
17 KS: 17-ketosteroid
17OHP: 17-hydroxyprogesterone
ACTH: Adrenocorticotrophic hormone
ALD: Serum aldosterone
CAH: Congenital adrenal hyperplasia
*CYP11B1mut*: Mutation p.L340P
DOC: 11-deoxycorticosterne
SNPs: Single nucleotide polymorphisms
