# Supplementary material for: A novel chimeric CYP11B2/CYP11B1 combined with a new p.L340P CYP11B1 mutation in a patient with 11OHD: case report
Source: BMC Endocr Disord. 2018 Apr 27;18:23. doi: 10.1186/s12902-018-0249-z (PMC5921981; doi:10.1186/s12902-018-0249-z)
Supplement: Supplementary file 1 — Table S1. The laboratory and endocrinological evaluation of the proband pre- and post-treatment. (PDF 200 kb) [file 12902_2018_249_MOESM1_ESM.pdf]

Supplemental Table 1 The laboratory and endocrinological evaluation of the proband pre- and post- treatment.

**Parameter**	**Diagnosis**	**Months after therapy**				**Reference Range**
		**4 months**	**9 months**	**20 months**	**28 months**	
**Laboratory examination**						
Potassium (mmol/L)	2.62	3.03	3.02	3.34	4.59	3.5-5.3
Natrium (mmol/L)	144.8	144.9	141.3	144.7	137.4	137-147
Chlorinum (mmol/L)	105.2	106.5	106.5	106.1	103.5	99-110
Urea (mmol/L)	2.55	3.94	2.42	NA	NA	2.9-8.2
Creatinine (mmol/L)	63	60.9	75.2	NA	NA	45-105
ALT (IU/L)	7	9.6	25	NA	NA	9-50
AST (IU/L)	18	16.4	16.5	NA	NA	15-40
TC (mmol/L)	2.65	NA	3.21	NA	NA	0-5.2
TG (mmol/L)	0.74	NA	0.88	NA	NA	0-1.7
Uric PH	7.5	7	NA	NA	NA	5.5-7.5
Plasma PH	7.37	NA	NA	NA	NA	6.5-7.45
pCO2 (mmHg)	45.7	NA	NA	NA	NA	35-45

Parameter	Diagnosis	Months after therapy				Reference Range
		4 months	9 months	20 months	28 months	
pO2 (mmHg)	99.8	NA	NA	NA	NA	83-108
UOP (mosm/kg)	317	437	314	NA	382	600-1000
USG	≤1.005	1.01	1.01	NA	NA	1.015-1.03
WDT UOP (mosm/kg)	394	NA	NA	NA	NA	600-1000
VT UOP (mosm/kg)	427	NA	NA	NA	NA	>429.46
**Endocrinological evaluation**						
ACTH (ng/L)	210	43.1	38.7	39.67	50.32	4.8-48.8
Cortisol (nmol/L)	205.8	237.8	193.9	167.9	21.4	176.6–579.4 (8 am)
	96.2	NA	NA	NA	NA	66–353 (4 pm)
	68.9	NA	NA	NA	NA	<100 (0 am)
17-KS (umol/24h)	98	45	NA	NA	NA	35-87
Aldosterone (pg/ml)	99.8	99.8	83.6	66	73.3	70-300
PRA (ng/ml/h)	0.02	0.02	0.37	0.03	1.15	0.1-6.56

Parameter	Diagnosis	Months after therapy				Reference Range
		4 months	9 months	20 months	28 months	
UVMA (mg/24h)	3.71	NA	NA	NA	NA	<13.6
MN (ng/L)	116.9	NA	NA	NA	NA	12.0-130.0
NMN (ng/L)	103.3	NA	NA	NA	NA	21.1-150.0
FSH (mIU/ml)	5.67	6.63	8.12	NA	NA	0.95–11.95
TEST (nmol/L)	19.73	22.46	38.18	NA	33.78	4.94-32.01
FT (pg/ml)	11.66	NA	10.41	NA	NA	0.20-42.50
DHEAS (ug/dl)	165.9	94.3	55.2	NA	NA	8.6-169.8
AD (ng/ml)	>10.3	7.98	4.36	NA	0.67	0.5-4.8
PROG (ng/ml)	2.7	2.1	0.3	NA	NA	<0.1–0.2
PRL (ng/ml)	8.63	8.64	5.65	NA	NA	3.46–19.4
E2 (pg/ml)	50	55	50	NA	56	11–44
LH (mIU/ml)	7.75	NA	NA	NA	NA	1.14–8.75
17-OHP (ng/ml)	18.05	6.24	5.51	NA	1.89	0.31-2.17
SHBG (nmol/L)	25.8	36.3	44.6	NA	NA	17.1-77.6

Parameter	Diagnosis	Months after therapy				Reference Range
		4 months	9 months	20 months	28 months	
FT3 (pmol/L)	4.81	5.99	3.78	NA	NA	3.1–6.8
FT4 (pmol/L)	18.64	17.74	16.5	NA	NA	11–22
TSH (mIU/L)	2.96	4.156	0.681	NA	NA	0.468–4.68

ALT alanine aminotransferase, *AST* aspartate aminotransferase, *TC* total cholesterol, *TG* triglyceride, *UOP* urine osmotic pressure, *USG* urine specific gravity, *WDT* water deprivation test, VT vasopressin test, *ACTH* adrenocorticotrophin, *17-OH* 17-hydroxycorticosteroid, *17-KS* 17-ketosteroid, *PRA* plasma renin activity, *UVMA* urinary vanillymandelic acid, *MN* metadrenaline, *NMN* normetadrenaline, *FSH* follicle-stimulating hormone, *TEST* testosterone, *FT* free testosterone, *DHEAS* dehydroepiandrosterone sulfate, *AD* androstenedione, *PROG* progesterone, *PRL* prolactin, *E2* estradiol, *LH* luteinizing hormone, *17-OHP* 17-hydroxy progesterone, *SHBG* sex hormone binding globulin, *FT3* free triiodothyronine, *FT4* free thyroxine, *TSH* thyroid-stimulating hormone, *PH* potential of hydrogen, *pCO2* partial pressure of carbon dioxide, *pO2* partial pressure of oxygen, *UOP* urine osmotic pressure, *USG* urine specific gravity, *WDT* water-deprivation test, *VT* vasopressin test.
