# Supplementary material for: A novel chimeric CYP11B2/CYP11B1 combined with a new p.L340P CYP11B1 mutation in a patient with 11OHD: case report
Source: BMC Endocr Disord. 2018 Apr 27;18:23. doi: 10.1186/s12902-018-0249-z (PMC5921981; doi:10.1186/s12902-018-0249-z)
Supplement: Supplementary file 2 — Table S2. Bioinformatics analysis of the free energy alteration in CYP11B1mut (PDF 82 kb) [file 12902_2018_249_MOESM2_ESM.pdf]

**Supplemental Table 2 Bioinformatics analysis of free energy alteration of CYP11B1mut**

| Software | Change of free energy (Kcal/mol) | Result |
|---|---|---|
| **mCSM** | -1.62 | Destablizing |
| **SDM** | -4.85 | Destablizing |
| **DUET** | -2.02 | Destablizing |
