# Supplementary figures and images for: A novel chimeric CYP11B2/CYP11B1 combined with a new p.L340P CYP11B1 mutation in a patient with 11OHD: case report
Source: BMC Endocr Disord. 2018 Apr 27;18:23. doi: 10.1186/s12902-018-0249-z (PMC5921981; doi:10.1186/s12902-018-0249-z)

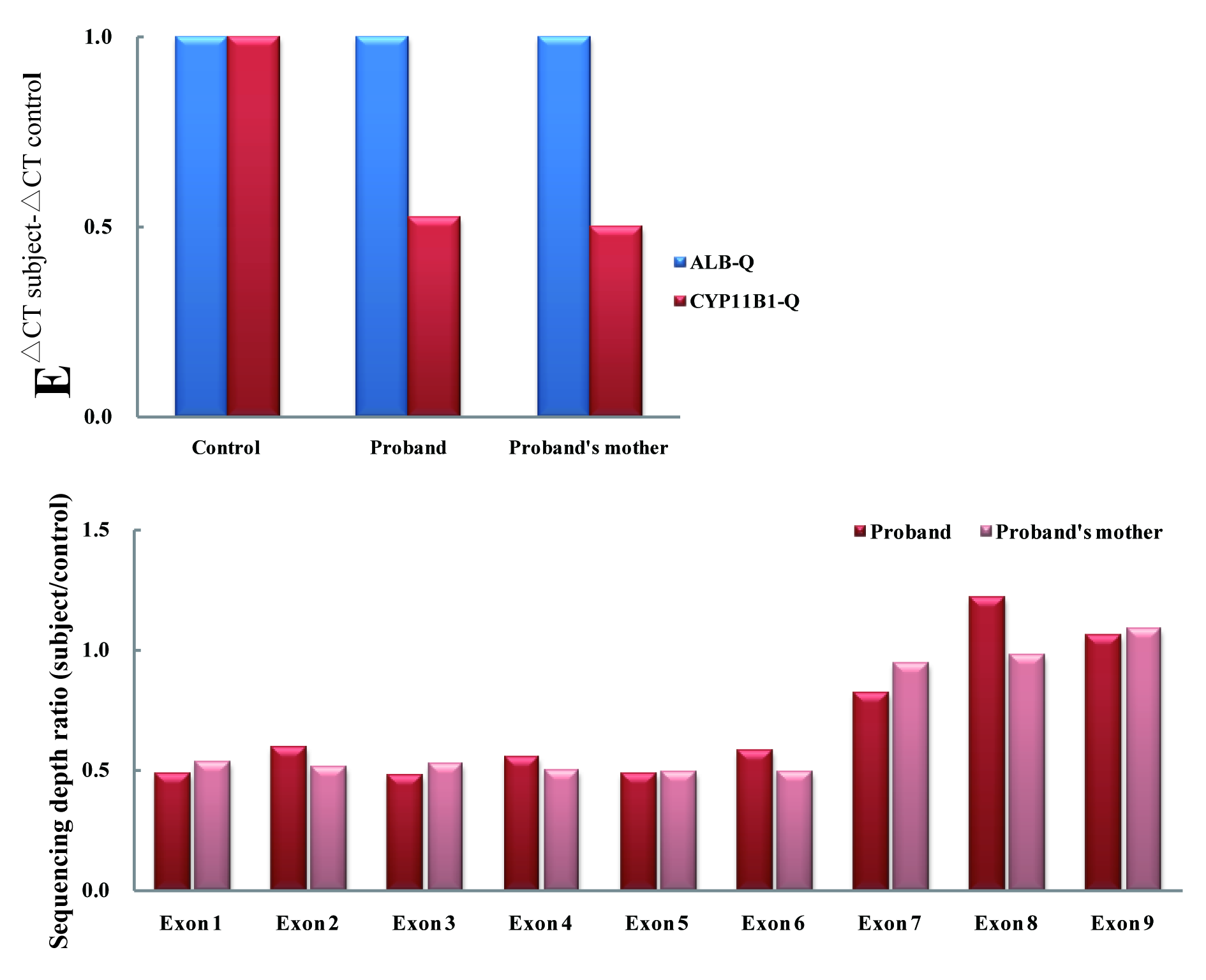

Supplement: Supplementary file 3 — Figure S1. Graphs of sequence copy number around the mutation and multiple genes resulting in CAH of the proband and his mother by qPCR and targeted next-generation sequencing. (TIF 11910 kb) [file 12902_2018_249_MOESM3_ESM.tif]

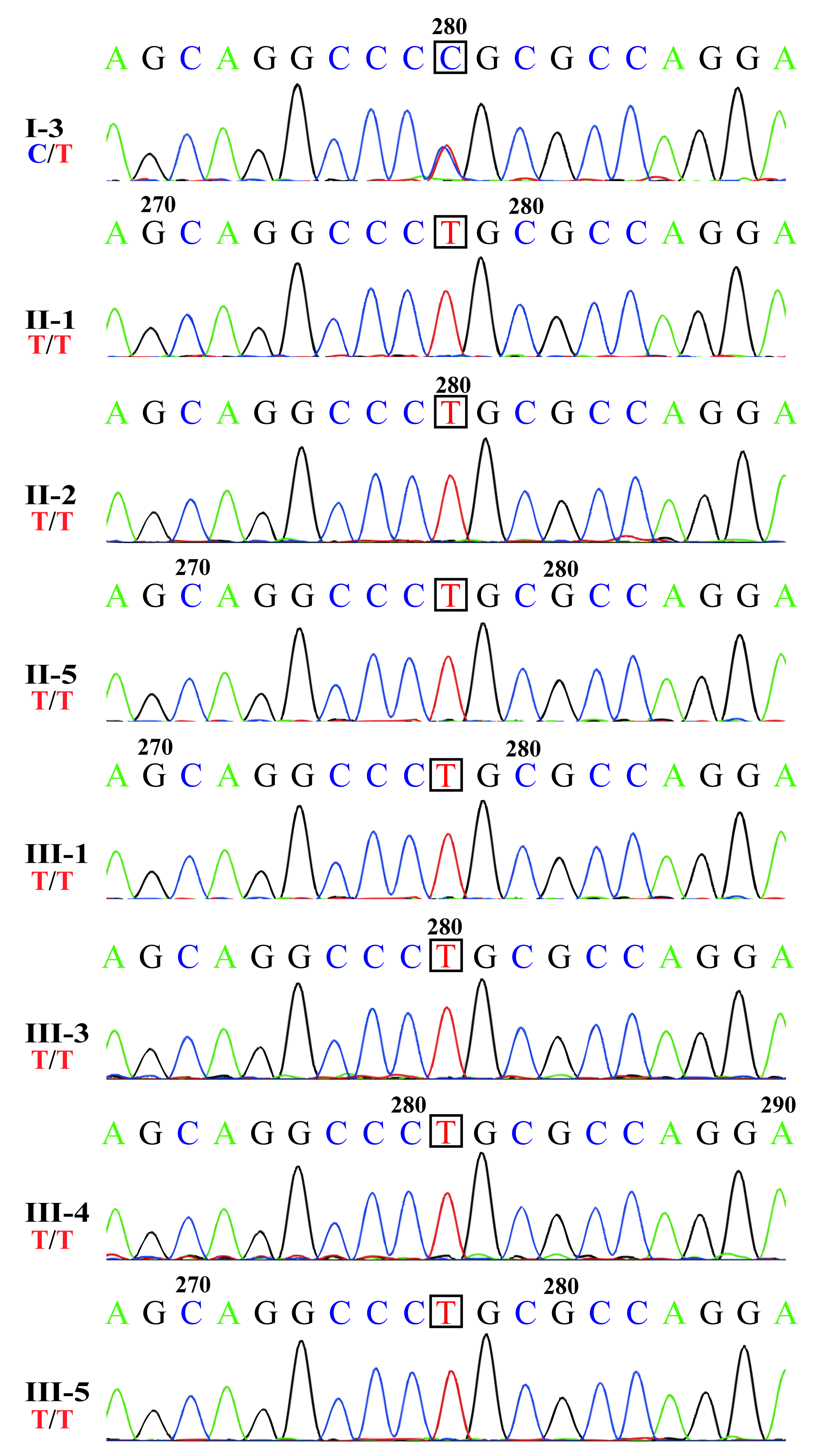

Supplement: Supplementary file 4 — Figure S2. The sequencing chromatogram near the mutation in the proband’s relatives. The box indicates the mutation location. (TIF 28380 kb) [file 12902_2018_249_MOESM4_ESM.tif]

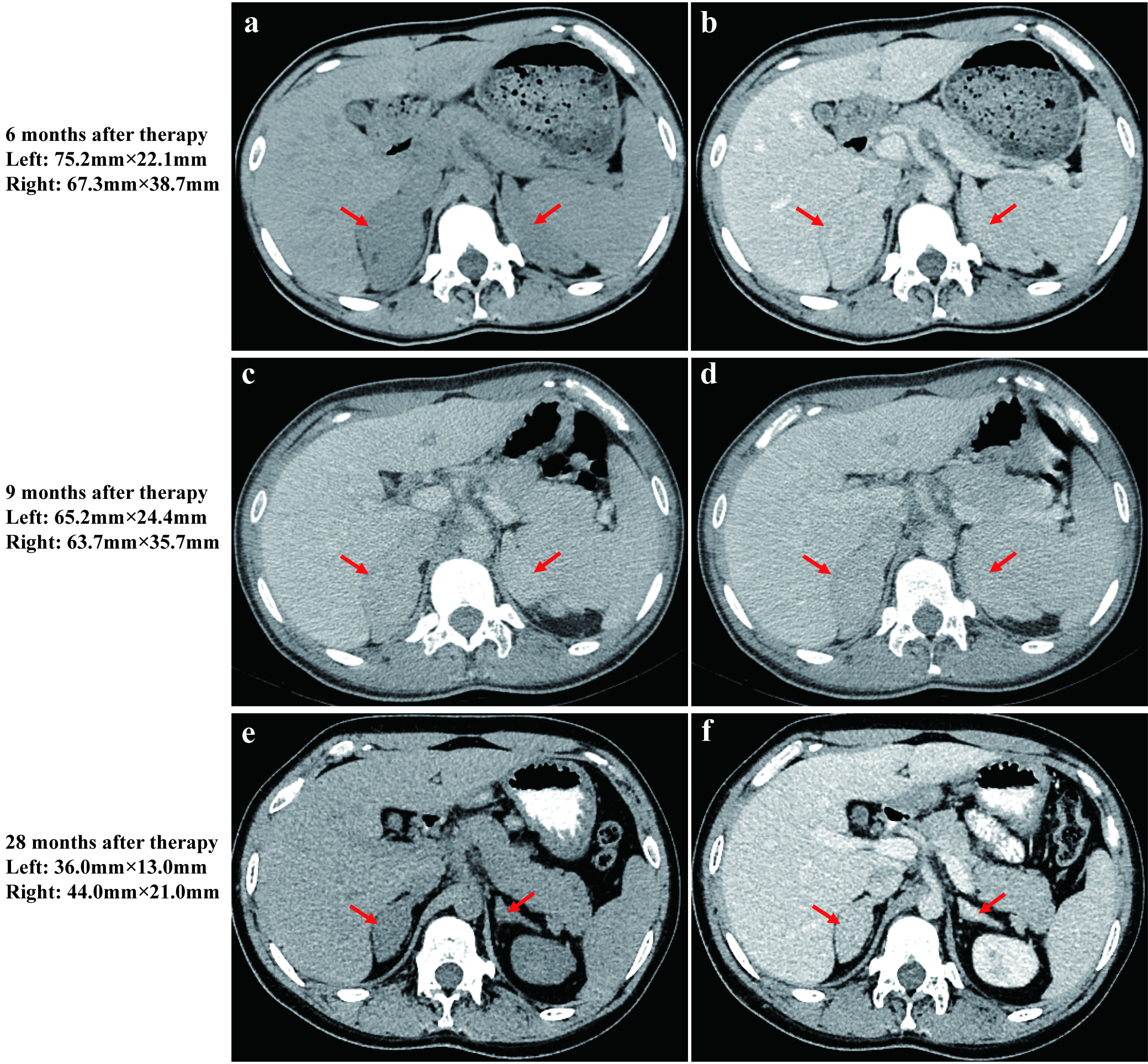

Supplement: Supplementary file 5 — Figure S3. Radiation imaging of adrenal gland after treatment. A and B present abdominal CT images after treatment for 6 months showing that the sizes of the left and right adrenal gland are 75.2 mm × 22.1 mm and 67.3 mm × 38.7 mm, respectively. Similarly, C and D present abdominal CT images after treatment for 9 months showing that the sizes of the left and right adrenal gland are 65.2 mm × 24.4 mm and 63.7 mm × 35.7 mm, respectively. The arrow shows the bilateral adrenal lesions. (TIF 63145 kb) [file 12902_2018_249_MOESM5_ESM.tif]
