# Supplementary material for: A novel chimeric CYP11B2/CYP11B1 combined with a new p.L340P CYP11B1 mutation in a patient with 11OHD: case report
Source: BMC Endocr Disord. 2018 Apr 27;18:23. doi: 10.1186/s12902-018-0249-z (PMC5921981; doi:10.1186/s12902-018-0249-z)
Supplement: Supplementary file 6 — Main methods used in the study. (DOC 112 kb) [file 12902_2018_249_MOESM6_ESM.doc]

**Methods**

**Laboratory and endocrinological evaluation**

Cortisol, FSH, TEST, FT, DHEAS, PROG, PRL, E2, SHBG, LH, aldosterone, angiotensin I and AD were measured using chemiluminescence immunoassays. TSH, FT4, FT3, and ACTH concentrations were measured using Rache electrochemiluminescence immunoassays. VMA and 17-KS were measured by chromatogram-spectrophotometry. MN and NMN were measured by high-performance liquid chromatography-electrochemical detection.

**DNA sequencing and Quantitative real-time PCR (QPCR)**

Genomic DNA was extracted from peripheral blood lymphocytes according to standard procedures using QIAamp DNA Bloodmini kits (Qiagen, Germany). We designed a targeted capture sequencing assay to test a panel of 154 metabolic disease genes (listed in the table at the end of the Methods), including the three known CAH-related genes (Mygenostics Co. Ltd., China). The targeted genes were enriched using a biotinylated capture probe (MyGenostics, Baltimore, MD, USA) as described previously . The enrichment libraries were sequenced using an Illumina HiSeq 2000 sequencer. The depths of each region of a gene within the same sequencing lane in different samples were significantly correlated (r > 0.7) and used to calculate a z-score with a predefined cut-off point (±3). Sanger sequencing using specific previously reported primers was used to confirm the mutations. The sequences generated from the patients were analyzed using Chromas 2.6.2 (Technelysium Pty Ltd, Australia) and compared with the reference sequence GenBank NG_007954.1 using Version 9 (Lynnon Biosoft, San Ramon City, CA, USA). CNV near the mutation was validated using qPCR on a 7900HT Fast Real-Time PCR system (Applied Biosystems, Foster City, CA, USA). One primer pair (forward: GCAAGGTCTGACCCTGCAGCTG and reverse: AAGGTCTCCTTGAGGGCCGCA) was designed using Primer Premier 5.0 software. The data were acquired using SDS 2.4 software (Applied Biosystems), and further relative quantification analysis was performed using the Pfaffl method while taking into account the amplification efficiencies . Meanwhile, PCR and Taq Big Dye Terminator Sequencing were conducted using a mixed oligonucleotide strategy following procedures previously reported with a pair of primers: sense 5’-TGTCCCCACTGGAAAGCTCT-3’complementary to the CYP11B2 sequence and antisense 5’-ACGCTCCTCACCATACCAAC-3’complementary to the CYP11B1 sequence.

**Bioinformatics and molecular dynamics simulation**

Using the MODELLER 9.11 package, the three-dimensional (3D) structure of wild-type and mutant 11β-hydroxylase protein was modeled using the crystal structure of human aldosterone synthase (PDB ID 4DVQ) as a template on the basis that it had the highest identity to 11β-hydroxylase (over 90%) . Position 340 is located in a J helix region on the surface of the protein, and exchange of leucine with proline was destabilizing and altered the free energy of the protein by over -1 kcal/mol, as predicted by DUET, SDM and mCSM software. Therefore, a 100 nanosecond molecular dynamics simulation of the wild-type and mutant proteins was performed using GROMACS package 4.5.4 as previously reported . To assess protein stability, the root-mean-square deviations (RMSD) for both the mutant and wild-type proteins were calculated, and the RMSD plots had relatively the same shape throughout the entire simulation; however, unlike wild-type, which was stabilized after 20 ns, the mutant showed a rising trend and higher variation in RMSD values at the end of the simulation.

**Table List of the 154 metabolic disease-related genes in the panel for targeted capture sequencing.**

| AASS | AR | CTH | FOLR2 | HPD | MOCS1 | PHGDH | SLC6A19 | ETFB |
| --- | --- | --- | --- | --- | --- | --- | --- | --- |
| ABAT | ARG1 | CYP11B1 | FOXG1 | HSD17B10 | MOCS2 | PNPLA2 | SLC6A20 | ETFDH |
| ABCD4 | ARX | CYP17A1 | FTCD | HSD17B3 | MTHFR | PNPO | SLC6A8 |  |
| ABHD5 | ASPA | CYP21A2 | G6PD | HSD3B2 | MTR | PRODH | SLC6A7 |  |
| ACAD8 | ASS1 | D2HGDH | GALK1 | INPP5E | MTRR | PSAT1 | SLC6A9 |  |
| ACADM | ATP7A | DBH | GAMT | IVD | MUT | PTPN11 | SOX9 |  |
| ACADS | ATP7B | DDC | GCSH | L2HGDH | MVK | PTS | SPR |  |
| ACADSB | AUH | DHFR | GCDH | LAMP2 | NAGS | QDPR | SRD5A2 |  |
| ACADVL | BCAT1 | DHTKD1 | GCH1 | MAOA | NR0B1 | SARDH | SRY |  |
| ACAT1 | BCAT2 | DLD | GLDC | MAT1A | NR5A1 | SERAC1 | StAR |  |
| ACSF3 | BCKDHA | ERCC6 | GLUD1 | MCCC1 | LMBRD1 | SLC19A1 | SUCLG1 |  |
| ADK | BCKDHB | ERCC8 | GLUL | MCCC2 | OAT | SLC22A5 | SUOX |  |
| AHCY | BTD | ASL | GPHN | MCEE | OGDH | SLC25A13 | TAT |  |
| ALDH4A1 | C7orf10 | DBT | HADHA | MECP2 | OPA3 | SLC25A15 | TAZ |  |
| ALDH5A1 | CBS | PAH | HADHB | MLYCD | OTC | SLC25A20 | TH |  |
| ALDH6A1 | CDKL5 | ETHE1 | HAL | MMAA | PC | SLC2A1 | UROC1 |  |
| ALDH7A1 | CPS1 | FAH | HGD | MMAB | PCBD1 | SLC2A2 | WT1 |  |
| ALPL | CPT1A | FH | HLCS | MMACHC | PCCA | SLC36A2 | SLC3A1 |  |
| AMT | CPT2 | FOLR1 | HMGCL | MMADHC | PCCB | SLC46A1 | ETFA |  |

**References**

1. An W, Zhang J, Chang L, Zhang Y, Wan Y, Ren Y *et al*: **Mutation analysis of Chinese sporadic congenital sideroblastic anemia by targeted capture sequencing**. *Journal of hematology & oncology* 2015, **8**:55.

2. Charnwichai P, Yeetong P, Suphapeetiporn K, Supornsilchai V, Sahakitrungruang T, Shotelersuk V: **Splicing analysis of CYP11B1 mutation in a family affected with 11beta-hydroxylase deficiency: case report**. *BMC endocrine disorders* 2016, **16**(1):37.

3. Pfaffl MW: **A new mathematical model for relative quantification in real-time RT-PCR**. *Nucleic acids research* 2001, **29**(9):e45.

4. Xu L, Xia W, Wu X, Wang X, Zhao L, Nie M: **Chimeric CYP11B2/CYP11B1 causing 11beta-hydroxylase deficiency in Chinese patients with congenital adrenal hyperplasia**. *Steroids* 2015, **101**:51-55.

5. Khattab A, Haider S, Kumar A, Dhawan S, Alam D, Romero R *et al*: **Clinical, genetic, and structural basis of congenital adrenal hyperplasia due to 11beta-hydroxylase deficiency**. *Proceedings of the National Academy of Sciences of the United States of America* 2017, **114**(10):E1933-E1940.

6. Agrahari AK, Sneha P, George Priya Doss C, Siva R, Zayed H: **A profound computational study to prioritize the disease-causing mutations in PRPS1 gene**. *Metabolic brain disease* 2017.

7. Margreitter C, Oostenbrink C: **MDplot: Visualise Molecular Dynamics**. *The R journal* 2017, **9**(1):164-186.
