# Supplementary material for: A novel chimeric CYP11B2/CYP11B1 combined with a new p.L340P CYP11B1 mutation in a patient with 11OHD: case report
Source: BMC Endocr Disord. 2018 Apr 27;18:23. doi: 10.1186/s12902-018-0249-z (PMC5921981; doi:10.1186/s12902-018-0249-z)
Supplement: Supplementary file 7 — Consent form from the mother of the proband. (PDF 95.7 kb) [file 12902_2018_249_MOESM7_ESM.pdf]

Consent form

For a patient's consent to publication of information about them in publications and products.

Name of person described in article or shown in photograph: Chunlan Zhang

Subject matter of photograph or article: A novel chimeric CYP11B2/CYP11B1 combined with a new p.L340P CYP11B1 mutation in a patient with 11OHD: case report

Journal name: *BMC endocrine disorders*

Manuscript number__BEND-D-17-00303__

Title of article: **A novel chimeric CYP11B2/CYP11B1 combined with a new p.L340P CYP11B1 mutation in a patient with 11OHD: case report**

Corresponding author: Hongting Zheng

I_______张春兰_______________ [insert full name] give my consent for

this information about MY CHILD [circle correct description] relating to the subject matter above ("the Information") to appear in the journal and associated publications.*

I have seen and read the material to be submitted to the journal

I understand the following:

(1) The Information will be published without my name attached and authors. I understand, however, that complete anonymity cannot be guaranteed. It is possible that somebody somewhere - perhaps, for example,somebody who looked after me if I was in hospital or a relative - may identify me.

(2) The text of the article will be edited for style, grammar, consistency, and length

(3) The Information may be published in the journal, which is distributed worldwide. The journal goes mainly to doctors but is seen by many non-doctors, including journalists.

(4) I can revoke my consent at any time before publication, but once the Information has been committed to publication ("gone to press") it will not be possible to revoke the consent.

Signed: 张春兰 Date: 2017.12.10
